# Undefining life's biochemistry: implications for abiogenesis

**DOI:** 10.1098/rsif.2021.0814

**Published:** 2022-02-23

**Authors:** Stephen Freeland

**Affiliations:** Department of Biological Sciences, University of Maryland Baltimore County, Baltimore, MD 21250, USA

**Keywords:** abiogenesis, evolution, central dogma, autocatalytic network, chemical evolution

## Abstract

In the mid-twentieth century, multiple Nobel Prizes rewarded discoveries of a seemingly universal set of molecules and interactions that collectively defined the chemical basis for life. Twenty-first-century science knows that every detail of this Central Dogma of Molecular Biology can vary through either biological evolution, human engineering (synthetic biology) or both. Clearly the material, molecular basis of replicating, evolving entities can be different. There is far less clarity yet for what constitutes this set of possibilities. One approach to better understand the limits and scope of moving beyond life's central dogma comes from those who study life's origins. RNA, proteins and the genetic code that binds them each look like products of natural selection. This raises the question of what step(s) preceded these particular components? Answers here will clarify whether any discrete point in time or biochemical evolution will objectively merit the label of life's origin, or whether life unfolds seamlessly from the non-living universe.

Last year, three publications described how the genetic material of more than 200 bacteriophage viruses uses 1-aminoadenine (Z) instead of adenine (A) [1–4]. This minor difference in chemical structures is nevertheless a fundamental deviation from the standard alphabet of four nucleobases established by biological evolution at the time of life's Last Universal Common Ancestor (LUCA). Placed into broader context, the finding illustrates a deep shift taking place in our understanding of the chemical basis for biology.

A slew of mid-twentieth-century Nobel Prizes were awarded for discovering a seemingly unifying molecular basis for all life on our planet. Nucleotide sequences (nucleic acid) carry each organism's genetic material, written in the alphabet of four nucleobases described above, whereas protein sequences, comprising the very different chemical language of amino acids, produce the catalysis of metabolism [5]. The chemical structure of nucleic acid explains replication, including the inheritance of variations and hence evolution by natural selection [6]. Each protein's shape and catalytic function derive from the sequences produced by linking together just 20 different types of amino acids [7]. Each protein sequence is specified by a corresponding gene sequence [8] through a genetic code that defines a meaning (translation) for every possible genetic code-word [9]. Collectively, these foundations of biochemistry became known as the Central Dogma of Molecular Biology [10] and more than one of the Nobel-winning scientists talked about having uncovered the ‘secret(s) of life’ [11,12]. While this pioneering research may not have searched for or found a formal definition of life, it presented one, for most practical purposes, to the molecular biology revolution [13] that it unleashed (figure 1*a*).

**Figure 1.**
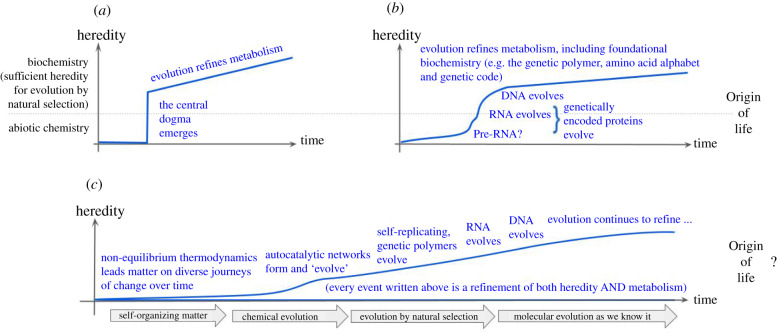
An evolving view of life's origins: from discontinuity to continuity. (*a*) Pioneering, mid-twentieth-century science that founded the molecular biology revolution [13] perceived a universal biochemical basis for life that implied a sharp discontinuity between abiotic chemistry and biological chemistry: life's origin was clearly the transition between these two, though further evolution of metabolism might refine the system of replication and evolution (e.g. protein enzymes that identify and correct genetic errors during replication). (*b*) By the start of the twenty-first century, it was clear that early evolution played a more significant role in establishing the central dogma than had been thought previously: DNA arrived in a world of RNA genes that encoded protein enzymes; RNA can and does take on functionality usually associated with protein enzymes; and both RNA and the standard genetic code appeared optimized relative to plausible chemical alternatives. (*c*) A current view adds the standard amino acid alphabet to the list of central dogma components, which appear optimized relative to plausible alternatives, along with strengthened evidence for RNA and the genetic code as outcomes of natural selection. Emerging insights about adaptive, evolutionary behaviour from collections of molecules far removed from nucleic acid sequences suggest that biological evolution by natural selection is a narrowed (refined) subset of broader processes by which matter (chemicals) change over time. Philosophically, this view aligns with calls to re-think life's emergence as a continuous process rather than any specific point in time or biochemistry. More practically, these same findings dissolve any clear distinction between the evolution of genetic versus metabolic aspects of life's biochemistry.

Fifty years later, the situation has changed. Z DNA merely complements prior discovery of extensions to the standard molecular alphabet of 20 amino acids [14] and different genetic codes connecting these two molecular languages [15] to blur chemical specifics for each central dogma component. Like ‘Z’ versus ‘A’, these naturally occurring variations are relatively minor, but it is unambiguous that life's molecular basis can and does evolve. Meanwhile, human ingenuity has successfully engineered into living organisms far more numerous and diverse alternatives to nucleobases [16,17], amino acids [18–20] and genetic codes [21]. In other words, progress in molecular biology has steadily undefined the chemical basis for life.

Flexible foundations to biochemistry carry direct implications for both ongoing synthetic biology and the search for extraterrestrial life. The successful development of a semi-synthetic organism, for example, which translates ‘ … a wide variety of unnatural ribonucleotides…to efficiently produce proteins containing multiple, proximal ncAAs [amino acids from beyond nature's standard genetic code]’ [17] demonstrates empirically that other collections of molecules than those found in life's central dogma are capable of sustaining evolving, self-replicating entities. More subtle, but arguably deeper implications arise for understanding abiogenesis when such flexibility is considered in the light of further studies which show collectively that each component of the central dogma resembles an outcome of natural selection. Compared with plausible alternatives, nucleobases appear to optimize the faithful transfer of genetic information [22,23] and their ribose/phosphate backbone optimizes the strength with which two strands of nucleic acid bind together [24]. The ‘alphabet’ of 20 amino acids represents unusually well the chemistry space available to this class of molecule [25], and the standard genetic code that binds these two molecular languages into a defined, functional relationship is one that appears to have been selected to minimize the deleterious impact of mutations [26,27].

If each component of the central dogma is an evolutionary upgrade of one or more preceding states then this redefines what abiogenesis requires from prebiotic chemistry. Illustrating the point, an authoritative 1981 review concluded that plausible prebiotic chemistry could account for most of life's standard amino acid alphabet and suggested that time and ingenuity would probably soon account for the rest [28]. Forty years later, multiple insights from different disciplines have converged upon a very different understanding: approximately one half of the standard alphabet was not a prerequisite for evolution, but invented by the evolution of novel biosynthetic pathways [29,30]. The twentieth amino acid, tryptophan, was not finalized until the time of LUCA [31]. This fresh perspective has already started to unlock unexpected, new insights about earlier phases of life's evolution. For example, the prebiotically plausible subset of amino acids form protein structures equally readily as the full alphabet but probably employing different mechanisms of compact structure formation [32].

Returning to genetic material, implications for abiogenesis are clear. So far, we know that DNA seems to have evolved within organisms using RNA genomes to encode proteins [33]. The discovery of catalytic RNA [34] erased any clean, functional distinction between protein catalysis and nucleic acid genes [34] and led to the RNA-world hypothesis which collapses the entire central dogma into a single chemical from which the central dogma later evolved [35,36] (figure 1*b*). This paradigm has caused some to perceive ‘a mandate for chemistry to explain how RNA might have been generated prebiotically on the early earth’ [37]. But if RNA, like half of the standard amino acid alphabet, evolved from one or more prior states then the perceived mandate changes. Seeking an origin for RNA within prebiotic chemistry becomes searching for prebiotically plausible molecules capable of evolving into RNA [38,39], quite possibly employing amino acids and/or other forerunners to modern metabolism along the way [40].

To be clear, many suggestions have been made for the identity of a possible evolutionary precursor to RNA [41,42] most have come from an RNA-world way of thinking, and none has gained consensus support, chemical exploration far beyond variations in structure or number of the central dogma's molecular components has been quietly developing ideas for a fundamentally different way to achieve self-replication. It has long been noted that ‘some non-covalent assemblies [of molecules] are capable of propagating their … compositional information without the involvement of long biopolymers such as RNAs or protein enzymes’ [43]. Within the many examples of autocatalytic networks of molecules that have been studied, however, the well-understood rules by which natural selection operates change, and a general framework to understand how is at best only now starting to emerge [44–46]. Critical re-analysis indicates that early findings were subtle artefacts of the model used [47], but it is not clear whether different modelling approaches can circumvent this problem and exploratory research continues. One intriguing suggestion is that we might usefully look to other systems of change over time that resemble some, but not all, the features of natural selection. For example, following an ‘instructive analogy between an autocatalytic cycle and biological species’ suggests that ‘chemical ecosystems can show complex dynamics that can resemble evolution’ [48].

Even further from post-LUCA biology, some physicists explore how non-living matter of all types can self-organize by dissipating free energy into ever more degrees of freedom. Whereas autocatalytic network research studies how ordered structures arise as equilibria that maximize entropy (i.e. minimizing Gibbs free energy), the physics of nonlinear, non-equilibrium thermodynamics describes a subtly different phenomenon: structures that arise spontaneously by increasing entropy in the surrounding environment. The idea that life's orderliness can arise as an imperative of energy dissipation traces back more than a century [49], although it drew much attention from Schrodinger's famous and profound lecture/monograph *What is Life?* [50] and earned yet another Nobel Prize (Chemistry, 1977 [51]) for the researcher who developed it into a precise mathematical framework [52]. This formation of dissipative structures certainly seems to describe at least some deep outcomes of biological evolution [53,54] and, perhaps, the prebiotic steps by which biological evolution itself emerged [55,56]. Sometimes (perhaps often) these energy-driven journeys of distinctly non-living matter terminate at outcomes that resist further change over time [57,58]. Carbon atoms can form diamonds or reactive organics, according to the environment in which they occur. We might call the former ‘dead-ends’ were it not for the danger of implying that they were achieving something life-like *en route*. More interesting for present purposes is the latter possibility: under the right conditions, pathways of change over time can lead matter into new configurations and conglomerations that are then capable of further change, under new rules [59,60]. To this kind of physics, the challenge is to understand what ‘physical conditions are most conducive to the emergence of novel self-replicating structures from a reservoir of building blocks on a desired time scale’ [61]. Illustrating just how much remains to be understood, one reviewer for this manuscript noted how importantly unclear remains the question of what exactly is meant here by ‘structures’. A blunt answer is that the word at present often describes abstract patterns of the model under scrutiny, such as interlocked square tiles [60]. The extent to which these abstract models extrapolate to account directly for the molecular foundations of post-LUCA biology is the all-important question [61].

Closing the gap between the molecular biochemistry that is leaking outwards from the confines of life's central dogma and the physics that is closing inwards from explorations of energy dissipation will require different research communities to better understand and appreciate one another's progress. This means transcending well-recognized challenges of interdisciplinary research [62]: different terminologies, different research forums and even different standards of evidence are currently at work. To a physicist studying nonlinear, non-equilibrium thermodynamics, chemical structure variations in the central dogma can easily seem hopelessly parochial for understanding the bigger picture of pathways that can produce biology. To a biologist studying chemical alternatives to RNA, differential equations that describe how matter behaves under a throughput of energy can easily seem over-generalized beyond anything of clear relevance. Better integration into a coherent, transdisciplinary picture of how life emerges therefore seems less likely to result from appropriately cross-trained individuals than from sustained effort to grow communication and collaboration between the existing research communities. It is therefore useful to consider just what is at stake. From an evolutionary perspective ‘The origin of life was the origin of true heredity … without heredity, and hence natural selection … usefulness [early shadows of metabolism] cannot begin’ [63]. Thus, a clear and discrete origin for life exists only if RNA (or a well-defined precursor) is necessary for the sort of unrestricted and open-ended inheritance that permits natural selection as we have come to understand and study that term within orthodox, evolutionary science. But with every step that RNA can be traced back to prior evolutionary state(s), the concept of abiogenesis begins to dissolve from any point defined clearly by biochemistry into a more continuous process by which life unfolds from the physics and chemistry of a non-living universe (figure 1*c*). If the right kind of matter changes over time into self-replicating chemical reaction networks by dissipating energy, and networks of the right kind can inherit compositional and/or structural features in a selection-like algorithm so as to eventually form RNA/protein metabolism, then it is not clear at what point such systems cross a line from physics and chemistry into biology.

Starting from the very different perspective of physical (bio)chemistry, the reasoning presented here thus aligns with deeper, philosophical arguments about meaningful definitions (or a lack thereof) for ‘life’. One recent contribution on this broader topic concludes that science has been ‘thinking incorrectly about the nature of life … finally abandoning the concept "life" may make our searches for evolved complexity more fruitful’ [64]. This argument in turn joins with a lineage of similar thought that has, for example, used ‘philosophical investigations into language to argue that defining "life" currently poses a dilemma analogous to that faced by those hoping to define "water" before the existence of molecular theory’ [65]. Such thinking has even offered practical suggestions for how embracing life's current, undefinable nature could ‘increase the likelihood of noticing truly novel forms of life’ in extraterrestrial exploration [66]. For some readers, that grand extrapolation might be the most interesting, potential interpretation of all that is written above: undefining life's biochemistry offers one more approach by which to question the (f)utility of seeking a definition for life. But the biochemical argument presented here is consciously narrower in scope. It limits consideration to how a loosened vision of life's chemical foundations might usefully progress the science of abiogenesis. This narrower focus finds its deepest and most deliberate alignment with another, singular philosophical contribution that challenged readers to rethink an older debate within origins research, namely the ‘conventional division between gene-first and metabolism-first groups’ [67]. A simple characterization of this debate sets one half of the central dogma (genetic information, usually in the form of an RNA-like polymer) against the other (the network of chemical reactions that sustain homeostasis, growth and reproduction, usually through protein catalysts) in an argument about which came first. Proponents of metabolism-first would identify easily with a notion of life seamlessly unfolding from within the non-living universe as they point to ancient metabolic processes, evolutionarily conserved to the present day, that closely resemble reaction networks occuring at strictly abiotic energy gradients [68], such as hydrothermal vents [69]. Genes-first proponents would counter that, however interesting such similarities might be, the only way in which a modular, carbon-based network of molecular catalysts could emerge emulating and improvising upon abiotic chemical reaction pathways would be through the process of natural selection, with all that implies about heredity and genetic information [63]. But just as Fry [67] pointed out, any characterization of a straightforward dichotomy (genes or metabolism?) is over-simplified. Even in contemporary cells, genetic polymers are quite clearly *not* disembodied, heritable information. The shape and other physical attributes of nucleic acid sequences are, for example, at the centre of current understanding for how present-day organisms regulate networks of gene expression [70]. The ‘non-informational’ features of RNA led Crick to foreshadow the RNA-world hypothesis [71] and these phenotypic, metabolic aspects are exactly what provide evidence that RNA appears optimized to its current role relative to plausible alternatives [22–24]. In evolutionary terminology, genotype carries inescapably intertwined aspects of phenotype, and that fact seems to point to an undiscovered evolutionary history for RNA (figure 1*c*). Conversely autocatalytic networks, notions of compositional inheritance and models of self-organizing matter all argue that what might look at first like metabolism can, in fact, present heritable information—aspects of genotype. From this perspective, a different interpretation of ongoing progress in undefining of life's biochemistry is that it resolves an outdated and over-simplified origins debate. Once we recognize with clarity the present (still imperfect) dichotomy between genes and metabolism as an outcome of considerable evolution, the answer to ‘genes-first or metabolism-first?’ becomes ‘both, but probably in neither of the chemical formats (structures) by which we understand such terminology today’. Viewed in this way, joining the dots between a search for forerunners to RNA all the way to general statements about necessary outcomes of energy dissipation is, among other things, a journey to discover how and why evolution might favour an increasingly clear, dichotomous split between genetic information and metabolism. Answers here deepen any current understanding for the process of abiogenesis.

## Data accessibility

This article has no additional data.
